# Channel Projection-Based CCA Target Identification Method for an SSVEP-Based BCI System of Quadrotor Helicopter Control

**DOI:** 10.1155/2019/2361282

**Published:** 2019-12-16

**Authors:** Qiang Gao, Yuxin Zhang, Zhe Wang, Enzeng Dong, Xiaolin Song, Yu Song

**Affiliations:** ^1^Tianjin Key Laboratory for Control Theory and Applications in Complicated Systems, Tianjin University of Technology, Tianjin 300384, China; ^2^Engineering Training Center, Tianjin University of Technology, Tianjin 300384, China

## Abstract

The brain-computer interface (BCI) plays an important role in assisting patients with amyotrophic lateral sclerosis (ALS) to enable them to participate in communication and entertainment. In this study, a novel channel projection-based canonical correlation analysis (CP-CCA) target identification method for steady-state visual evoked potential- (SSVEP-) based BCI system was proposed. The single-channel electroencephalography (EEG) signals of multiple trials were recorded when the subject is under the same stimulus frequency. The CCAs between single-channel EEG signals of multiple trials and sine-cosine reference signals were obtained. Then, the optimal reference signal of each channel was utilized to estimate the test EEG signal. To validate the proposed method, we acquired the training dataset with two testing conditions including the optimal time window length and the number of the trial of training data. The offline experiments conducted a comparison of the proposed method with the traditional canonical correlation analysis (CCA) and power spectrum density analysis (PSDA) method using a 5-class SSVEP dataset that was recorded from 10 subjects. Based on the training dataset, the online 3D-helicopter control experiment was carried out. The offline experimental results showed that the proposed method outperformed the CCA and the PSDA methods in terms of classification accuracy and information transfer rate (ITR). Furthermore, the online experiments of 3-DOF helicopter control achieved an average accuracy of 87.94 ± 5.93% with an ITR of 21.07 ± 4.42 bit/min.

## 1. Introduction

Brain-computer interface (BCI) is a direct communication-control system which establishes a transmission channel between electrical signals of a human's brain and external devices without the involvement of muscles and peripheral nervous system [1]. For several decades, BCI techniques have been increasingly developed by utilizing neurophysiological signals, such as EEG, magnetoencephalography (MEG), near-infrared spectroscopy (NIRS), and functional magnetic resonance imaging (fMRI) [2–4]. Recently, EEG-based BCI has been successfully used in clinical rehabilitation, assistive mobility, mental-state recognition, and game due to its noninvasiveness, reliability, portability, and remarkable time signal resolution [5]. Moreover, BCI can not only improve the quality of life of disabled people but also can provide additional help and entertainment mode for healthy people to achieve multifunctional augmentative and alternative tasks [6].

Nowadays several basic paradigms have been utilized to realize EEG-based BCIs, such as event-related potential (ERP) [7], P300 potential [8], steady-state visual evoked potential (SSVEP) [9, 10], slow cortical potential (SCP) [11], and motor imagery (MI) [12]. SSVEP-based BCI has attracted much attention due to its possibility of achieving a high-dimensional control (degrees of freedom) which remains a critical issue for developing multidimensional and multifunctional BCI applications. In addition, the high signal-to-noise ratio (SNR), high ITR, and little training required can be achieved by the SSVEP-based BCI system [13]. The SSVEP is a periodic response of brain which is reflected by repetitive visual stimuli flickering with a certain fixed frequency (generally higher than 6 Hz). Several studies have been proposed to develop SSVEP-based BCI applications. The 40-target SSVEP-based BCI was developed for the high-speed brain speller, which can achieve an ITR of 267 bit/min [14]. An SSVEP-based BCI application was designed for a maze game. The four operation instructions, up, down, left, and right, were responded to four commands of stimuli to control the movement of an object in a maze [15]. The two different types of visual stimuli, pattern-reversal checkerboard stimulus (PRCS) and grow/shrink stimulus (GSS), were compared during SSVEP-based BCI application in virtual reality environment. It indicated that the optimal visual stimulus for an individual can improve the performance of the SSVEP-based BCIs and reduce visual fatigue in the VR environment [16]. The signal-channel SSVEP-based BCI speller system was designed [17]. The novel virtual keyboard contained 58 characters, special symbols, and digits, and the five stimulation boxes (6 cm × 6 cm) were present in each layer (three layers for one target character). The online experiment accuracy is about 97.4% with the ITR of 49 ± 7.7 bit/min.

However, for real-life applications, the multichannel system could not be widely accepted due to the high cost of the device and a complicated setup process. The commercial and low-cost EEG recording device, Emotiv EPOC, which combines low spatial resolution and acceptable signal quality, was used for BCI applications out of the lab. The Emotiv EPOC was utilized in the shooting game in which the subject could use their brain to control the direction of the pistol in the online target shooting [18]. The researchers designed a wearable BCI system based on SSVEP, which enabled 3-D navigation of quadcopter flight with immersive first-person visual feedback using a head-mounted device [19]. Therefore, the Emotiv EPOC device is quite enough to be used in portable daily-life applications to improve life quality for healthy and disabled people [20].

To improve the performance of SSVEP-based BCIs, the two main directions are taken into consideration [21]: increasing the number of stimuli classes and improving the accuracy by target recognition algorithms. The number of frequencies that can be presented on the computer screen is limited by its refresh rate. Hence, to improve the control performance and increase the number of selections, the hybrid modality that combines SSVEP and other EEG features (P300) or electrophysiology features has been developed [22]. Target recognition algorithms play an important role in enhancing the performance of SSVEP-based BCIs. The power spectrum density analysis (PSDA) is a widely utilized and easily implemented method in SSVEP recognition, which estimates the PSD values of EEG signals at different frequencies within a specific time window, typically by fast Fourier transform (FFT) and discrete Fourier transform (DFT) [23]. The frequency with the maximal PSD value (peak subsequently) is recognized as the visual stimulus frequency. Nevertheless, the PSDA is sensitivity to noise that leads to low accuracy in the SSVEP frequency detection. In addition, a relatively long time window is usually required to estimate the spectrum that may restrict its real-time application. To overcome the limitations of PSDA, several studies have employed multivariate statistical analysis as classifiers to detect SSVEP frequencies, such as canonical correlation analysis (CCA) [24] and least absolute shrinkage and selection operator (LASSO) [25]. The CCA-based classifier has been used to improve the classification accuracy in the cases of multichannel-based application due to its ability to enhance the SNR of the SSVEP-based BCI system [26]. The EEG data are multidimensional, which contain differences of multiple experiments, uncertainty among subjects, and so on. However, the above algorithms cannot satisfy the need for simultaneous processing of multidimensional information in EEG signals (especially SSVEP). In order to solve this limitation, we try to combine multidimensional signal processing technology with CCA algorithm to optimize and improve the performance of the BCI system in the process of feature extraction and classification of SSVEP.

This study proposed a channel projection-based target recognition method with CCA to improve the performance of an SSVEP-based BCI. The performance of an offline experiment was evaluated using a 5-target SSVEP dataset recorded from 10 subjects. The traditional CCA method and PSDA method were used to compare the performance with the proposed method. After optimizing parameters (data length, the number of training trials, and the number of electrodes), an online BCI system was carried out to control a 3-DOF helicopter by the proposed method. The structure of the remaining parts of the paper is as follows: Section 2 describes all details of the used materials, proposed classification/control methods, and offline and online experimental setups. The offline and online experimental results are shown out in Section 3. Finally, discussion and conclusion are presented in Sections 4.

## 2. Materials and Methods

**Figure 1:**
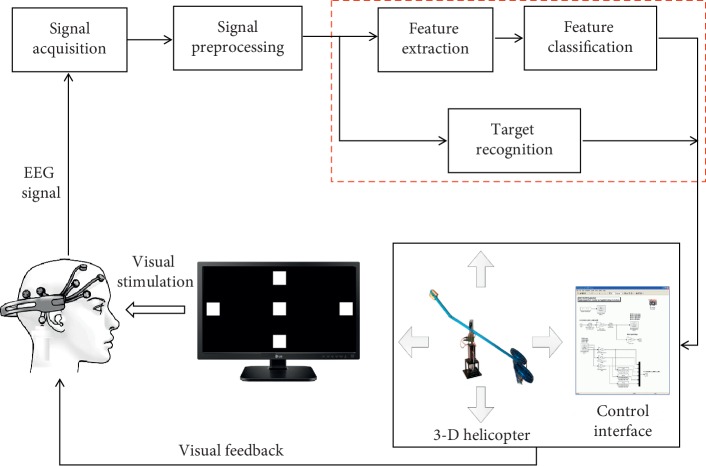
Block diagram of the proposed BCI system.

Figure 1 shows a block diagram of the proposed SSVEP-based BCI system. The subject sits on the chair in front of a liquid crystal display (LCD) screen and stares at the stimuli boxes. The raw EEG data are recorded by the dry electrodes and then are transmitted to the host computer for preprocessing to increase the SNR. For target recognition, one way is to combine the feature extraction method and feature classification method to find the right stimulus frequency. Another is using the different target recognition methods to identify the target stimuli. Finally, the control commands are generated by the computer according to the classification results. The 3-D helicopter will conduct the control commands to move to the target position.

### 2.1. Experiment Environment

Ten healthy volunteers (7 males and 3 females) participated in the offline and online experiments, respectively. All participants ranged in age from 21 to 26 (average age 24). These fully BCI-naive subjects have normal or corrected to normal vision. All the participants were informed by clear written consent about the purpose and possible consequences of the experiment in detail.

**Figure 2:**
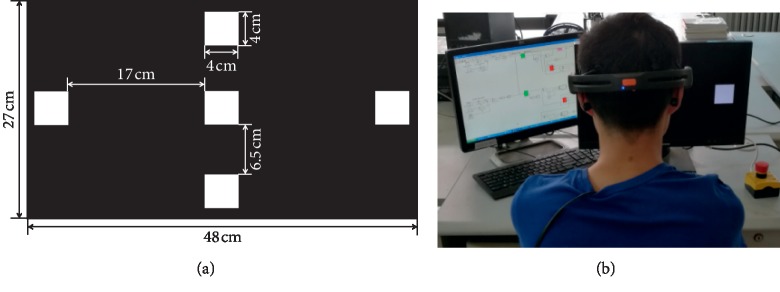
Stimulus design of 5-target BCI system. (a) The 5 stimuli boxes; (b) the control process.

In this BCI experiment, LCD was used to demonstrate stimulus on the monitor that the resolution and refresh rate are 1920 × 1080 pixels and 60 Hz, respectively. The black-white color combination was selected in the stimulator design to show different stimulus frequency. Each stimuli box is a square of 4 cm × 4 cm, as shown in Figure 2(a). The stimulus frequencies of the five targets are 6.67 Hz, 7.5 Hz, 8.57 Hz, 10 Hz, and 12 Hz which located at the left, middle, right, top, and bottom of the screen, respectively.

**Figure 3:**
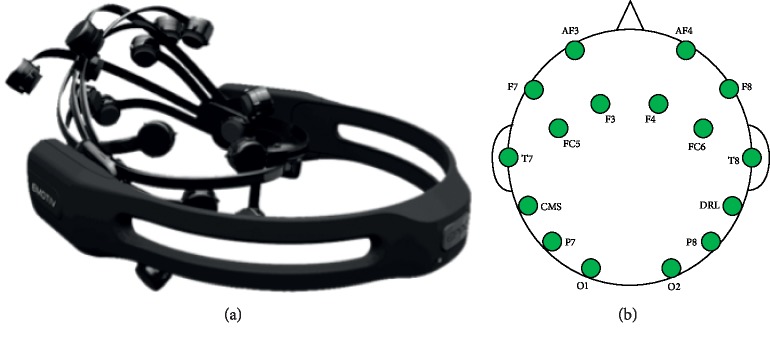
EEG acquisition device. (a) Emotiv EPOC; (b) electrode position according to 10–20 EEG placement.

Combined with the cost-effective, portable, and no training features, Emotiv EPOC headset is used to collect EEG signals, and the device is shown in Figure 3. For brain activity recording, 14 channels are placed on the standard locations according to the 10–20 international system, which are named AF3, F7, F3, FC5, T7, P7, O1, O2, P8, T8, FC6, F4, F8, and AF4. Moreover, CMS/DRL reference positions are also employed, which are located behind the ear of the subject. The EEG signals are sampled at an internal rate of 2048 Hz and down-sampled to 128 Hz in each EEG channel.

### 2.2. Experimental Design

#### 2.2.1. Offline BCI Experiment

**Figure 4:**
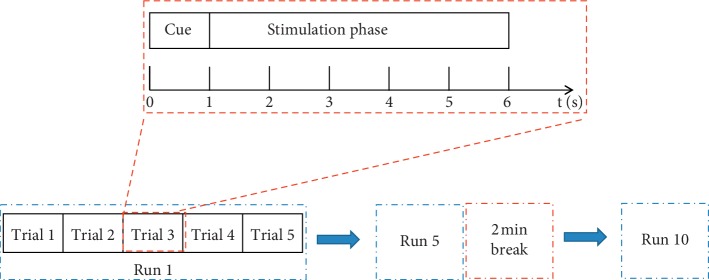
The timing of the whole procedure.

During the SSVEP-based BCI experiment, the subjects seated in a comfortable position in a normally bright room, with a 40 cm distance from the monitor, which was placed in front of the subjects. For the offline experiment, the subjects performed a simulated online experiment to record EEG data for offline analysis. The subjects were guided to gaze one of the five stimulus targets according to the command that was sounded by the speaker. Each subject completed 10 runs, and each run was composed of 5 trials. To prevent the subjects from visual fatigue, the 2 min break was given after 5 runs. Moreover, every stimulus frequency was performed with a random sequence. Each trial lasted 6 seconds and consists of two parts: a cue phase with 1 s and a stimulation phase with 5 s.  Figure 4 shows the timing of the whole procedure. To reduce eye movement artifacts, subjects were asked to avoid eye blinks during the stimulation. The ten-fold cross-validation was utilized to evaluate the precision of SSVEP recognition for one subject. That means nine trails as the training dataset, and then the rest one trial was the testing data. The traditional CCA method and PSDA method were used to compare the performance with the proposed method.

#### 2.2.2. Online BCI Experiment

**Figure 5:**
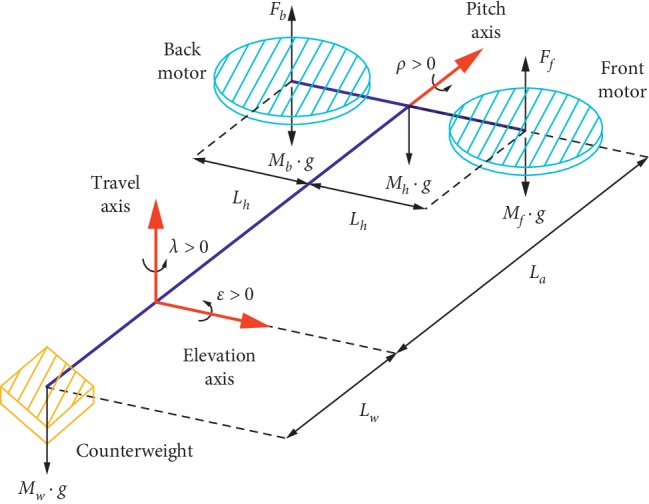
The mathematical model of the 3-DOF helicopter.

**Figure 6:**
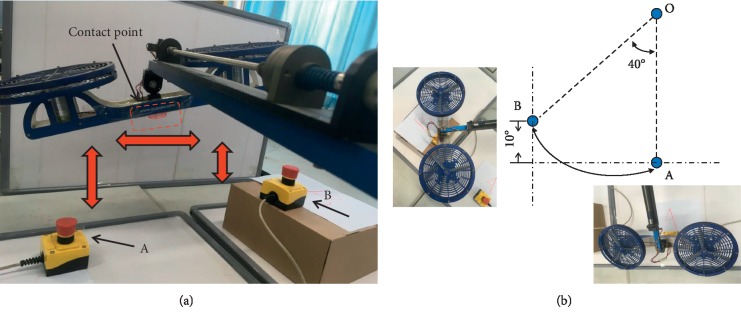
The top view of the helicopter flight task. (a) Flight experimental setup; (b) location information and motion curve, the contact point moves from position A to position B and then goes back to position A, and the moving step is 10 degrees in four directions (up, down, left, and right).

The online experiment was conducted to validate the effectiveness of the proposed feature recognition method. The offline data were utilized as a training dataset during the online experiment. The subject was asked to control a 3-DOF helicopter by the proposed method. The schematic of the helicopter is shown in Figure 5. The state-space equation of the linearized system was utilized in our previous study [27]. The Matlab toolboxes and QuanRC software were used to connect the experiment platform for controlling the attitude of the 3-DOF helicopter. According to designed stimulus frequencies, four tasks of the helicopter can be conducted (left, right, upward, and downward movements) which correspond to four stimuli positions on the screen. The middle stimuli box of the screen is used to cancel the previous action when it comes with a wrong command. The top view of the helicopter flight task is shown in Figure 6. The subject was required to navigate the helicopter from the start position A to the target position B and then went back to position A. When the helicopter received one control command, it would move ten degrees along the axis in the corresponding reorientation. Thus, 18 right commands should be produced to finish the task. The number of correct counts of the 18 commands is used to evaluate the system accuracy. Once the incorrect command is generated, the user can choose the middle box to take the helicopter back to the last position.

To evaluate the overall system, the classification accuracy and ITR were calculated. The ITR is a well-known parameter for BCI system evaluation [28]. For a trial with *N* possible targets in which each target has the same possibility, the classification accuracy *P* that the target will be hit is the same for each target. The higher ITR means that the BCI system can transfer more information per unit of time. The bits of information communicated per one minute were calculated as follows:

(1) $$\begin{array}{c} R=\frac{60}{T}\log_{2}N+P\;\log_{2}P+\left(1-P\right)\log_{2}\left(\frac{1-P}{N-1}\right), \end{array}$$

where *T* represents the time window length. If the value of *N* is fixed, the ITR is only affected by the value of *T* as well as by the value of *P*. In this study, the number of targets is 5, and the range of time window length is from 1 s to 5 s.

### 2.3. Target Recognition Algorithm

#### 2.3.1. SSVEP Recognition Based on CCA

The CCA method is able to calculate the underlying correlation between two multidimensional data. Therefore, CCA extends the ordinary correlation to two sets of random variables and has been widely used in the recognition of SSVEPs [24, 29]. In other words, the CCA aims to find a pair of linear transformations, which called canonical variants, for two sets of multidimensional variable, so as to achieve the maximum correlation between the two canonical variants. Suppose that two multidimensional random variables **X** and **Y** (**X** ∈ *R*^*h*×*i*^ and **Y** ∈ *R*^*j*×*i*^). CCA finds a pair of weight vectors *w*_*X*_ ∈ *R*^*h*×1^ and *w*_*Y*_ ∈ *R*^*j*×1^, respectively, which maximize the correlation between linear combinations *x*=*w*_*X*_^*T*^**X** and *y*=*w*_*Y*_^*T*^**Y**. It is defined as

(2) $$\begin{array}{c} \underset{w_{X},w_{Y}}{\max}\rho \left(x,y\right)=\frac{E\left[xy^{T}\right]}{\sqrt{E\left[xx^{T}\right]E\left[yy^{T}\right]}}=\frac{E\left[w_{X}^{T}XY^{T}w_{Y}\right]}{\sqrt{E\left[w_{X}^{T}XX^{T}w_{X}\right]E\left[w_{Y}^{T}YY^{T}w_{Y}\right]}}, \end{array}$$

where max*ρ* is the maximum canonical correlation. *x* and *y* are projected onto *w*_*X*_ and *w*_*Y*_. Therein, **XX**^*T*^ and **YY**^*T*^ are the within-sets covariance matrices, and **XY**^*T*^ is the between-sets covariance matrix. Each CCA leads to a number of solutions max*ρ* equal to the minimum between the number of rows in **X** (*h*) and **Y** (*j*). The solutions max*ρ* are a measure of the similarity between the two sets of original data.

To distinguish the *m* stimulation frequencies, the CCA will be performed *m* times. For a certain stimulation frequency *f*_*k*_(*k*=1,  2,…,  *m*), the CCA between the multichannel EEG signal in **X** (*h* presents the number of EEG channels, and *i* is the number of sampling points in each channel) and a reference signals in **Y**_*i*_ is calculated. **Y** is the reference signal that is artificially generated with sine and cosine waves at the stimulus frequency *f*_*k*_, and *j* is the number of harmonics. The reference signals are set as

(3) $$\begin{array}{c} Y_{i}=\left(\begin{array}{c} \sin\left(2\pi f_{k}t\right)\\ \cos\left(2\pi f_{k}t\right)\\ \vdots \\ \sin\left(2\pi jf_{k}t\right)\\ \cos\left(2\pi jf_{k}t\right) \end{array}\right),\;t=\frac{1}{s},\frac{2}{s},\ldots ,\frac{i}{s}, \end{array}$$

where *s* is the sampling rate. The brain dynamics plays a low-pass filter, and the high harmonic components in a square wave may be filtered. The four harmonics was used in this work. The correlation coefficients between the EEG signal and different reference signal is calculated by (3). As a result, the target frequency *f*_*s*_ is recognized as

(4) $$\begin{array}{c} f_{s}=\underset{f_{k}}{\max}\rho_{k},\;k=1,\;2,\ldots ,\;m. \end{array}$$

#### 2.3.2. Channel Projection-Based Target Recognition Method with CCA

Although the powerful performance of the CCA-based method in detecting SSVEP has been proved by researchers [24, 26, 30]. However, the detectability of SSVEP with different frequencies can be influenced by the power-law distribution of the power spectra spontaneous electroencephalogram (EEG) signals. Thus, CCA may not give best accuracy for SSVEP classification, especially in using a relatively short time window. Several studies have tried to alleviate this problem. An unsupervised method is reported to derive normalized canonical correlation coefficients for CCA to enhance the frequency detection of SSVEP. Zhang et al. proposed the MCCA [31] and L1-MCCA [32] methods to optimize sine-cosine reference signals by correlating the multiple dimensions of EEG signals. Then, the common features were used as reference signals instead of sine-cosine signals to improve recognition accuracy. The core idea of those methods is using multiple trials that the subject focuses attention on the same visual stimuli to get a reference signal through the training procedure, which can reduce the inherent differences of the subject.

**Figure 7:**
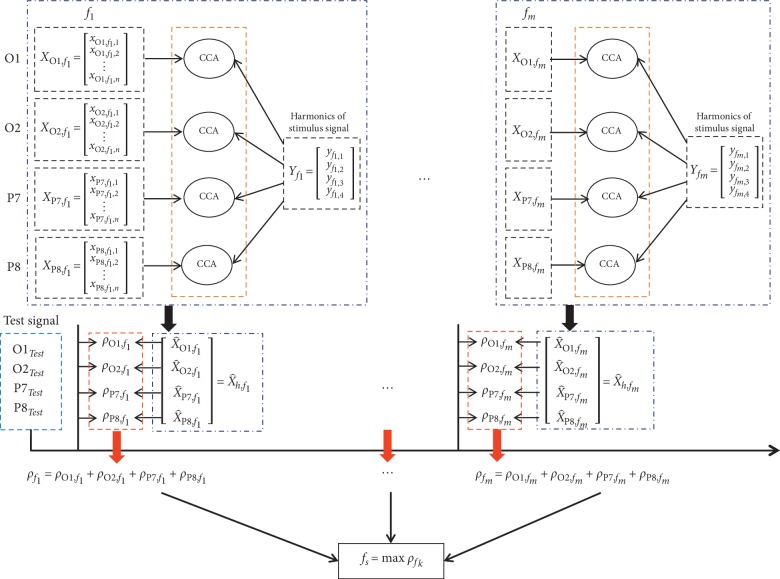
The process of the proposed CP-CCA (channel projection-based CCA) for SSVEP target recognition. For the same target frequency  *f*_*m*_, the different trials of recorded EEG data are dispersed and then reorganized according to the different channels, **X**_O1,*f*_*k*__, **X**_O2,*f*_*k*__, **X**_P7,*f*_*k*__, and **X**_P8,*f*_*k*__. The optimal reference signals of different channels (${\hat{X}}_{\text{O}1,f_{k}},{\hat{X}}_{\text{O}2,f_{k}},{\hat{X}}_{\text{P}7,f_{k}},$ and ${\hat{X}}_{\text{P}8,f_{k}}$) under certain stimulus frequency *f*_*k*_ are obtained by the CCA between the channel-based EEG data and the sine-cosine signals **Y**_*f*_*k*__. The SSVEP target frequency *f*_*s*_ of a new test data of single trial is recognized according to the maximum value of the sum of *ρ*_*h*,*f*_*k*__.

Inspired by the above study, we utilized the CCA method to find optimal data to represent the multiple trials of EEG data that were recorded by the single channel when the subject gazed at the same frequency of visual stimuli.  Figure 7 illustrates the flowchart of the proposed CP-CCA (channel projection-based CCA). Suppose that recorded EEG data of multitrials in the specific stimulus frequency are **X**_*h*,*f*_*k*__ ∈ *R*^*n*×*i*^, *n* is the number of trials, and *h* represents four different channels (O1, O2, P7, and P8). Here two vectors *w*_*h*,*x*_ ∈ *R*^*n*×1^ and *w*_*h*,*y*_ ∈ *R*^*j*×1^ are selected to find the maximum correlation coefficient of ${\hat{X}}_{h,f_{k}}=w_{h,x}^{T}\times X_{h,f_{k}}$ and ${\hat{Y}}_{f_{k}}=w_{h,y}^{T}\times Y_{f_{k}}$. The maximum correlation of one channel can be described as

(5) $$\begin{array}{c} \underset{w_{h,x},w_{h,x}}{\max}\rho_{h}=\frac{E\left[{\hat{X}}_{h,f_{k}}{\hat{Y}}_{f_{k}}^{T}\right]}{\sqrt{E\left[{\hat{X}}_{h,f_{k}}{\hat{X}}_{h,f_{k}}^{T}\right]E\left[{\hat{Y}}_{f_{k}}{\hat{Y}}_{f_{k}}^{T}\right]}}=\frac{E\left[w_{h,x}^{T}X_{h,f_{k}}Y_{f_{k}}^{T}w_{h,y}^{T}\right]}{\sqrt{E\left[w_{h,x}^{T}X_{h,f_{k}}X_{h,f_{k}}^{T}w_{h,x}^{T}\right]E\left[w_{h,y}^{T}Y_{f_{k}}Y_{f_{k}}^{T}w_{h,y}^{T}\right]}}. \end{array}$$

The reference signal ${\hat{X}}_{h,f_{k}}$ reflects the frequency component of SSVEP of different channels. Moreover, it contains the common character of the single channel with multitrials for the same stimulation frequency. When optimal reference signals of different stimulus frequencies ${\hat{X}}_{h,f_{1}},{\hat{X}}_{h,f_{2}},\ldots ,{\hat{X}}_{h,f_{m}}$ were obtained, the correlation coefficient *ρ*_*h*,*f*_*k*__ between the test signal and reference signal of the single channel (O1, O2, P7, and P8) can be calculated. The new test data of a single trial are recognized according to the maximum value *ρ*_*f*_*k*__, which is the sum of *ρ*_*h*,*f*_*k*__, and can be defined as

(6) $$\begin{array}{c} \rho_{f_{k}}=\rho_{\text{O}1,f_{k}}+\rho_{\text{O}2,f_{k}}+\rho_{\text{p}7,f_{k}}+\rho_{\text{p}8,f_{k}}. \end{array}$$

In this work, the number of target stimulation frequency *m* = 5. For the reference signal, its fundamental and second frequency components are considered in this design.

## 3. Results

### 3.1. Offline Experimental Results

**Figure 8:**
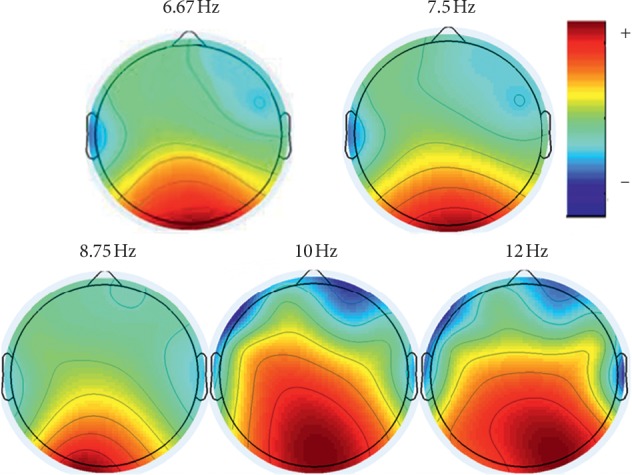
The brain frequency power map of subject 1 under 5 different stimulus frequencies.

The offline experiment aims to find the optimal parameter for online SSVEP recognition. In this offline analysis, the optimal time window length and the number of training data are discussed.  Figure 8 shows the brain frequency power map of subject S1 of one run. The red color represents the brain activity is markedly intense. On the contrary, the blue denotes parts of the brain that do not show significant activity.

**Figure 9:**
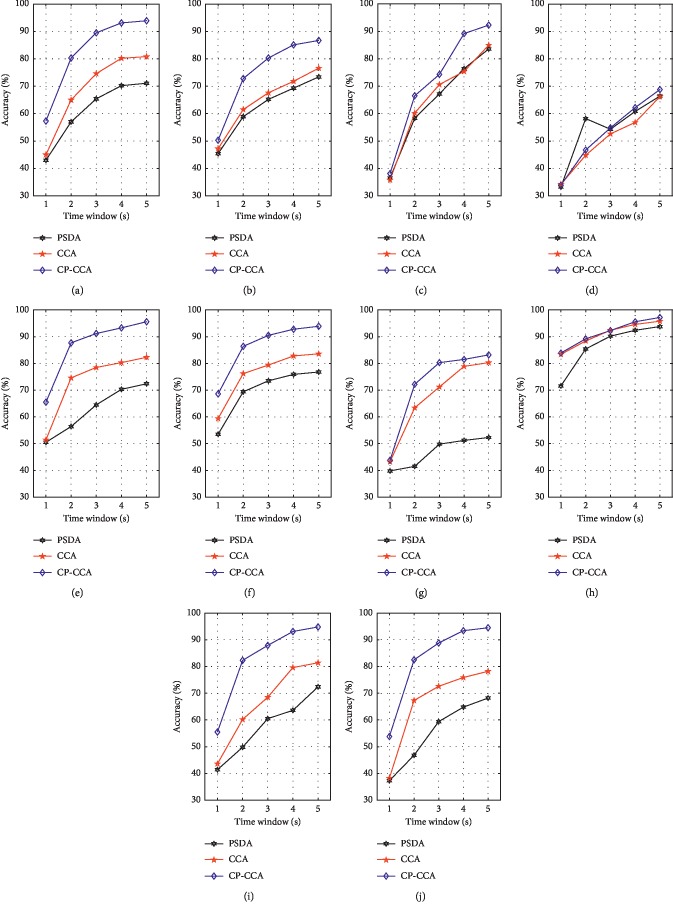
The averaged detection accuracy for each of ten subjects obtained by the PSDA, CCA, and proposed CP-CCA with respect to time window length from 1 s to 5 s. (a) S1. (b) S2. (c) S3. (d) S4. (e) S5. (f) S6. (g) S7. (h) S8. (i) S9. (j) S10.

Figure 9 shows the averaged detection accuracy for each of ten subjects obtained by the PSDA, CCA, and proposed CP-CCA with respect to time window length from 1 s to 5 s. The accuracy was estimated by 10-fold cross validation, in which 9 trials were used as training data, respectively, and 1 trial was used as test data. The results indicated that the classification accuracy was increasing with the stimulus time.

**Figure 10:**
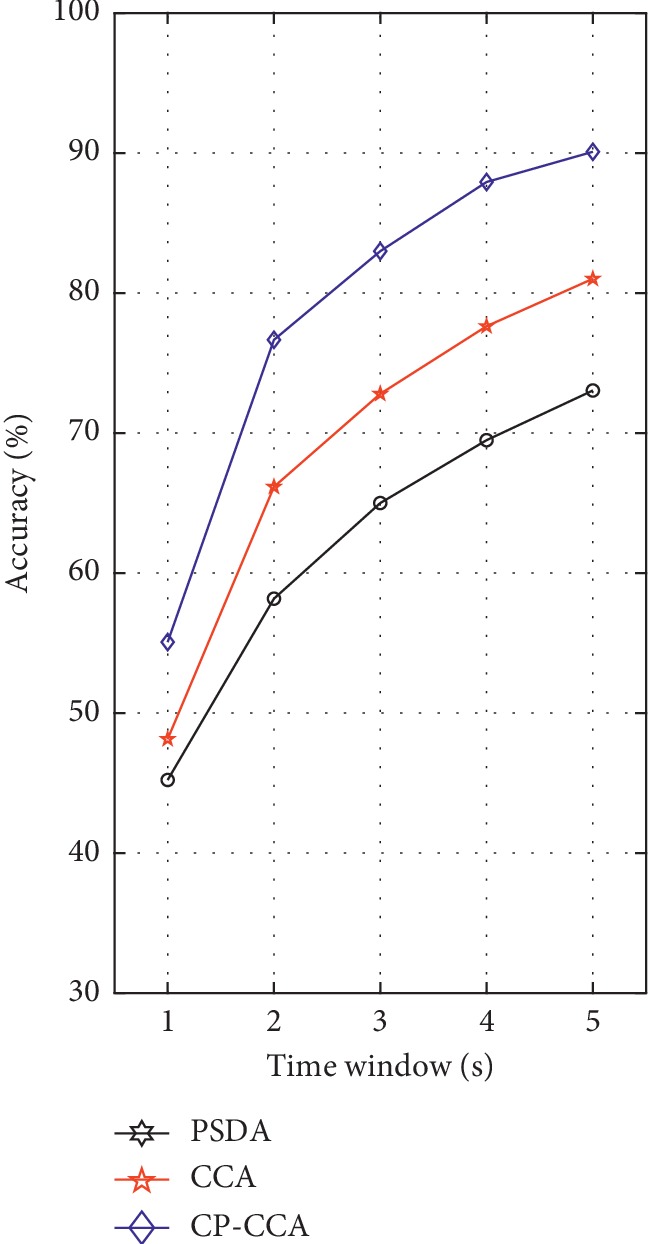
The average accuracy of all subjects by the PSDA, CCA, and proposed CP-CCA with respect to time window length from 1 s to 5 s.

Figure 10 depicts the average accuracy of all subjects by the three methods. These results demonstrate that the proposed CP-CCA significantly outperformed the PSDA and CCA for SSVEP-based target recognition at time window from 1 s to 5 s. The highest classification accuracy of the proposed CP-CCA was 90.09% for 5 s time window, whereas CCA and PSDA methods achieve their highest accuracy of 81.02% and 73.04%, respectively, in the case of 5 s window length.

**Figure 11:**
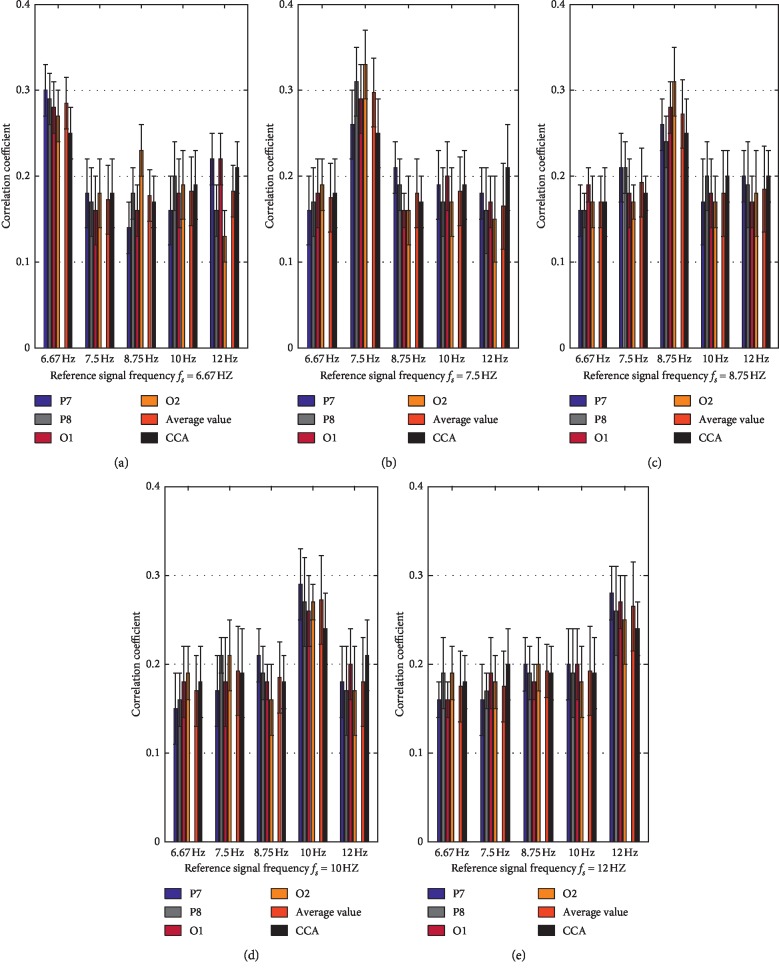
The correlation coefficients corresponding to different reference signal frequencies (6.67 Hz, 7.5 Hz, 8.57 Hz, 10 Hz, and 12 Hz) derived from 10-fold cross-validation by the CP-CCA (average value) and CCA at 5 s time window, when each of the five stimulus frequencies was used as the target frequency *f*_*s*_.

Figure 11 shows the correlation coefficients of each channel (P7, P8, O1, and O2) and the averaged value of four channels corresponding to different reference signal frequencies (6.67 Hz, 7.5 Hz, 8.57 Hz, 10 Hz, and 12 Hz) derived from 10-fold cross validation by the proposed CP-CCA at 5 s time window, when each of the five stimulus frequencies was used as the target frequency. In addition, these results were compared with the CCA-based method in the same experimental condition. From the results, compared to the CCA, the proposed CP-CCA achieved a higher correlation coefficient (average value of four channels) for the target frequency.

**Figure 12:**
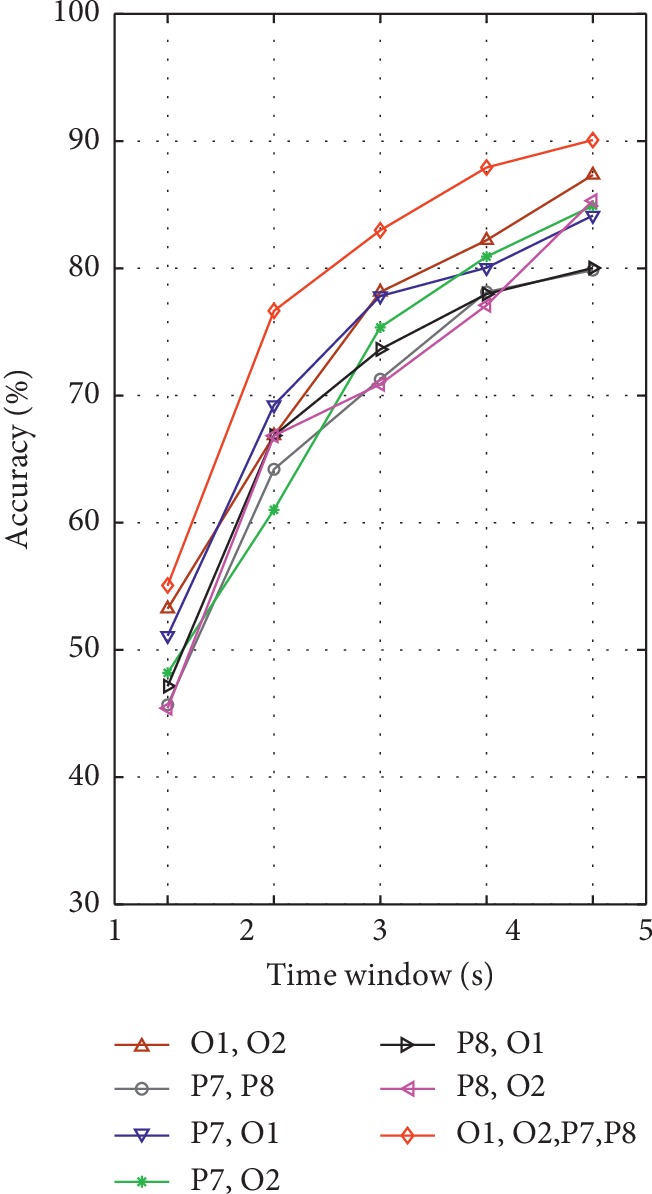
Comparison of detection accuracy of the different combination of electrodes placement with respect to time window length by the proposed CP-CCA method.

In addition, another analysis was carried out to find the optimal channel montage, and the result is shown in Figure 12. The accuracy was estimated by 10-fold cross-validation. The experimental results indicated that the combination of O1 and O2 has higher accuracy than the combination of P7 and P8 in five stimulus frequencies with different time window length but lower than the combination of O1, O2, P7, and P8. The results also demonstrated that, in most cases, the combination of the ipsilateral electrodes has better results than the combination of the contralateral electrodes. The combination of O1 and O2 makes a significant contribution to the SSVEP detectability. Finally, we choose four channels (P7, P8, O1, and O2) as a combination, due to their higher classification accuracy.

**Figure 13:**
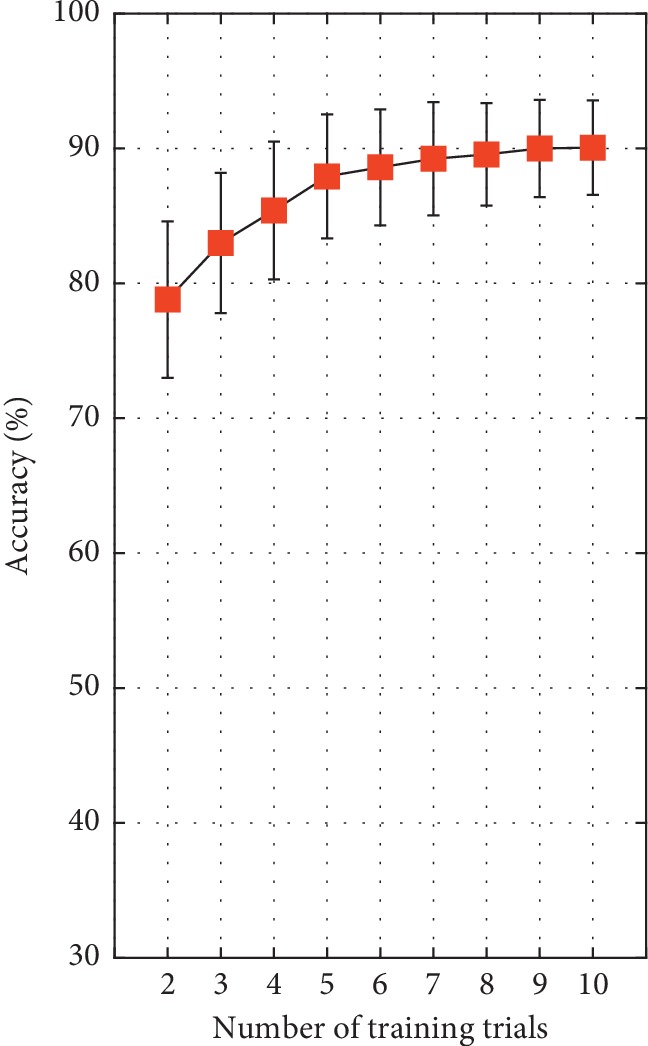
The average classification accuracy with different numbers of training trials for all subjects in terms of 5 s window length.

Figure 13 shows the average classification accuracy of all subjects with the number of training trails (from 2 to 10) using 5 s window length. The highest accuracy of 90.09% was obtained when the numbers of training trials were 10. In addition, when the number of training trial was 5, the classification accuracy is over 89%.

### 3.2. Real-Time Experimental Results

For ensuring both detection accuracy and ITR, during the online experiment, the selected time window is 4 s. The 0.5 s interval was given to subjects to shift their gaze between the 3-D helicopter and stimuli boxes.  Table 1 lists the results of the online experiment of the 3-DOF helicopter control across 10 subjects. 4 training trials of the offline experiment were utilized for each of the 5 stimulus frequencies. The average accuracy of the proposed control strategy over all subjects was 87.94 ± 5.93%, and the average of the commands was 23.60 ± 3.24 bit/min. All subjects achieved accuracy of over 80%. Moreover, subject 1 successfully completed the control task without any mistake. The average ITR is 21.07 ± 4.42 bit/min.

## 4. Discussion and Conclusion

In this work, we aimed to develop a practicable SSVEP-based BCI system by Emotiv EPOC considering real-life feasibility. The target identification algorithm plays an important role in the improvement of the performance of the SSVEP-based BCI system since other elements such as stimulus design and the number of electrodes should also be taken into consideration. To evaluate the effectiveness of the proposed CP-CCA target identification method, we compared its performance with the traditional CCA and PSDA methods. As shown in Figure 10, the proposed method obtained higher classification accuracy than CCA and PSDA for all time windows. During this study, the single channel EEG signals of multiple trials were recorded when the subject is under the same stimulus frequency. The CCAs between single-channel of multitrials EEG signals and sine-cosine reference signals were obtained. The optimal reference signal contains the common character of the single channel with different trials under the same stimulus frequency. Figure 11 provides evidence for the superior SSVEP-based recognition accuracy over the CCA. The use of CP-CCA may solve the limitation of interference from the spontaneous EEG activities and reduce the inherent differences of the subject.

The proposed CP-CCA requires individual training data before the online BCI control experiment. The number of training trials is an important parameter in target reorganization. As shown in Figure 13, the classification accuracy increased with the number of training trials. However, for a convenient and efficient online BCI system, the no training or few training times is essential [13]. From the offline experimental results, the classification accuracy of the 5-fold cross validation over 89% was achieved. In addition, reminding that an increase in window length may cause a decrease in ITR, an increase in the time of each command, and increase in the total time to complete a task. For ensuring both detection accuracy and ITR, 4 s time window was selected for online experiment. The experiment results from 10 subjects in controlling the 3-DOF helicopter showed that the averaged accuracy was 87.94 ± 5.93% with an ITR of 21.07 ± 4.42 bit/min. The online averaged accuracy was slightly lower than the offline that using the same number of training trials. The difference might be influenced by the experimental conditions between the online and offline modes, such as SNR of the EEG signal light conditions in the room, subjects' visual fatigue, and subject's awareness of 3-DOF helicopter control. Some studies showed that spatial filtering techniques were effective to remove EEG artifact, leading to increasing SNR and SSVEP classification [24, 33]. Another factor was the low resolution in accuracy during the online experiment (100% means that the subject continuously executes 18 right commands). In addition, the result from the low sampling frequency and fewer useful channels for SSVEP target recognition of Emotiv EPOC, the classification accuracy, and ITR of the designed system are limited. Some studies utilized 8 or 9 channels for SSVEP target recognition by the CCA-based method. However, in real-life applications, a multichannel system could be an inefficient device because of its complicated setup. The convenience of the Emotiv EPOC which increases the usability as well as reduce the complexity of the system while maintaining the wearing comfort over time may make up the shortcoming.

In this paper, we proposed a novel channel projection-based CCA target identification method for the SSVEP-based BCI system with a portable device. The offline analysis results showed that the proposed method outperformed the CCA and PSDA methods in terms of classification accuracy. The online application was validated by the 3-DOF helicopter control experiment. 10 subjects achieved an average accuracy of 87.94 ± 5.93% with an ITR of 21.07 ± 4.42 bit/min. By using the low-cost EEG acquisition device, this study will encourage more real-life BCI applications for communication and control in assisting people with disabilities. An automated system is a key feature for efficient BCI control. Further studies will be performed to develop programmable program to reduce the total number of commands and improve the flexibility and practicability of the system for disabled.

## Figures and Tables

**Table 1:** Results of the online 3-DOF helicopter control experiments.

Subject	No. of commands	Accuracy (%)	ITR (bit/min)
S1	18	100.00	30.96
S2	24	87.50	20.38
S3	20	95.00	25.81
S4	28	82.14	17.17
S5	24	87.50	20.38
S6	22	90.91	22.68
S7	26	84.61	18.59
S8	28	82.14	17.17
S9	28	82.14	17.17
S10	24	87.50	20.38
Mean ± STD	23.60 ± 3.24	87.94 ± 5.93	21.07 ± 4.42

## Data Availability

The [.txt] data used to support the findings of this study are available from the corresponding author upon request.
